# The Atypical Cobalamin-Dependent *S*-Adenosyl-l-Methionine Nonradical Methylase TsrM and Its Radical Counterparts

**DOI:** 10.1021/jacs.1c12064

**Published:** 2022-03-28

**Authors:** Emily C. Ulrich, Catherine L. Drennan

**Affiliations:** ^†^Department of Biology and ^‡^Department of Chemistry, Massachusetts Institute of Technology, Cambridge, Massachusetts 02139, United States; § Howard Hughes Medical Institute, Massachusetts Institute of Technology, Cambridge, Massachusetts 02139, United States

## Abstract

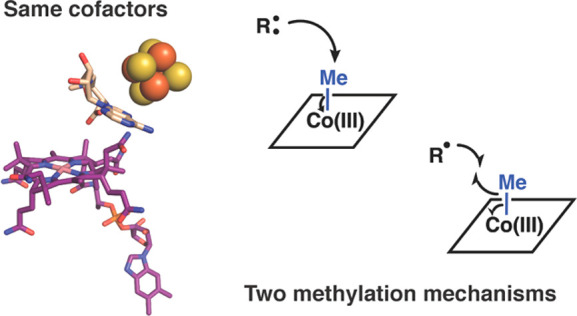

Cobalamin (Cbl)-dependent *S*-adenosyl-l-methionine (AdoMet) radical methylases are known for their use of a dual cofactor system to perform challenging radical methylation reactions at unactivated carbon and phosphorus centers. These enzymes are part of a larger subgroup of Cbl-dependent AdoMet radical enzymes that also perform difficult ring contractions and radical rearrangements. This subgroup is a largely untapped reservoir of diverse chemistry that requires steady efforts in biochemical and structural characterization to reveal its complexity. In this Perspective, we highlight the significant efforts over many years to elucidate the function, mechanism, and structure of TsrM, an unexpected nonradical methylase in this subgroup. We also discuss recent achievements in characterizing radical methylase subgroup members that exemplify how key tools in mechanistic enzymology are valuable time and again. Finally, we identify recent enzyme activity studies that have made use of bioinformatic analyses to expand our definition of the subgroup. Additional breakthroughs in radical (and nonradical) enzymatic chemistry and challenging transformations from the unexplored space of this subgroup are undoubtedly on the horizon.

## Introduction

The *S*-adenosyl-l-methionine (AdoMet or SAM) radical enzyme superfamily performs difficult chemical transformations on a variety of substrates including small molecules, intricate natural products near the final stage of synthesis, and amino acids within a large protein.^1^ The superfamily label is applicable to this enzyme group given that its membership is estimated to be over 100 000 enzymes from all domains of life, which is significant growth from its initial establishment with 650 members in 2001.^2,3^ The list of members is still expanding through continual genome sequencing and enzyme characterization. This superfamily can be broken into numerous subgroups that cover a breathtaking array of chemical reactions, such as carbon skeleton rearrangements, oxidations, and methylations at *sp*^2^- and *sp*^3^-hybridized carbon centers.^1^

**Figure 1:**
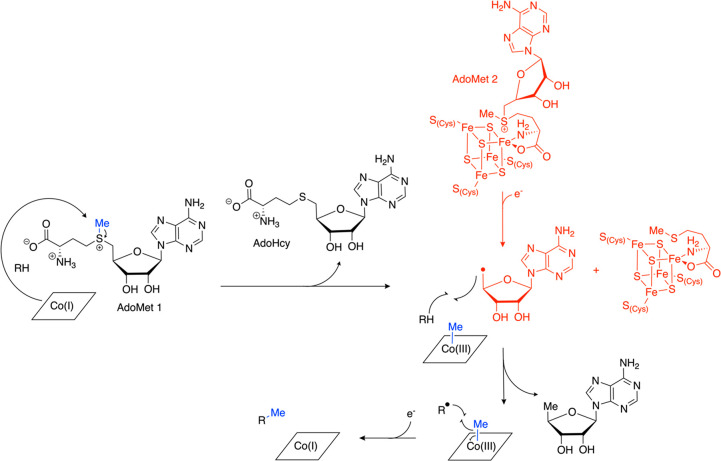
A standard methylation mechanism proposed for Cbl-dependent AdoMet radical methylases with a generic “RH” substrate. One AdoMet is used to methylate cob(I)alamin and another AdoMet participates in radical chemistry to generate a substrate radical (shown in red). A methyl group from methylcob(III)alamin is then transferred to the substrate radical via homolytic cleavage of the cobalt–carbon bond. The electrons required for [4Fe-4S] cluster and cob(II)alamin reduction can be provided by a flavodoxin/flavodoxin reductase/NADPH reducing system in vitro and are potentially provided by this system in vivo as well.

**Figure 2:**
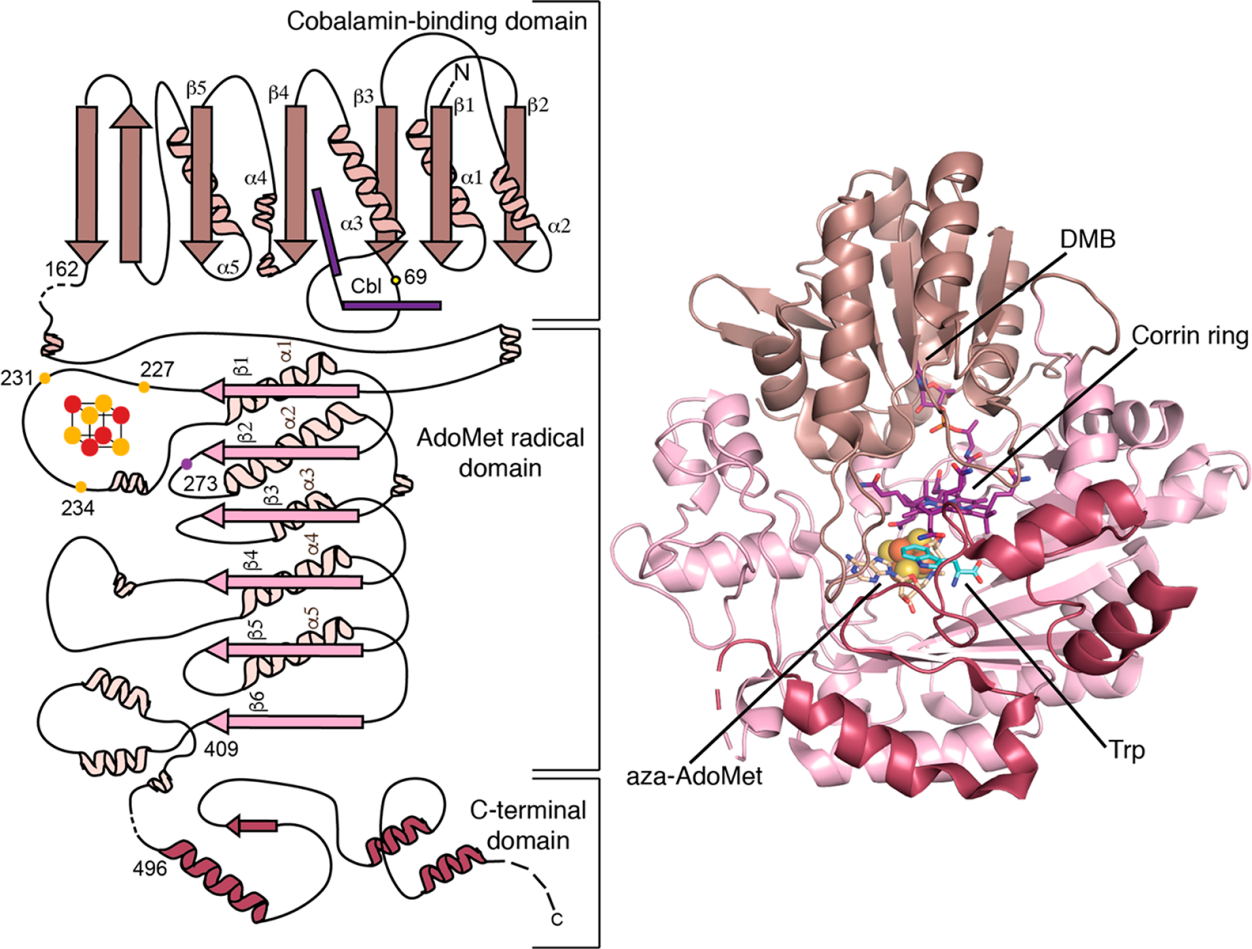
Topology diagram and overall structure of TsrM revealing the separate domains required for two cofactor machineries. The Cbl-binding domain (brown) sits N-terminal to the AdoMet radical domain (light pink) and consists of a Rossmann fold and a displaced dimethylbenzimidazole (DMB) lower ligand to the cobalt. The AdoMet radical domain is followed by a C-terminal domain (raspberry) made up mostly of helices. Dashed lines indicate disordered regions of the structure. The [4Fe-4S] cluster is in orange and yellow, Cbl is in deep purple, aza-AdoMet is in wheat, and Trp is in aquamarine. TsrM PDB ID: 6WTF

AdoMet radical enzymes have defining structural and mechanistic features, although exceptions are accumulating as new enzymes are discovered and characterized.^1,4^ The AdoMet radical cofactor is comprised of a molecule of AdoMet coordinated via its carboxylate and amino groups to the unique iron of a [4Fe-4S] cluster (Figure 1).^5−7^ The standard radical mechanism kicks off with the one electron reduction of the [4Fe-4S] cluster and homolytic cleavage of the carbon–sulfur bond of the coordinated AdoMet, resulting in methionine (Met) and a 5′-deoxyadenosyl radical^8^ (5′-dAdo•, see reaction in red in Figure 1). Recent studies by Broderick and Hoffman^9,10^ have suggested that formation of 5′-dAdo• involves an intermediate (called omega) that has an organometallic bond between the 5′ carbon of the deoxyadenosine and the unique iron of the [4Fe-4S] cluster. Omega has not been characterized crystallographically, but the formation of an alkyl-[4Fe-4S] species has been demonstrated by spectroscopic methods in a number of AdoMet radical enzymes^9^ and in a model system recently developed by the Suess lab.^11^ Once formed, 5′-dAdo• can abstract a hydrogen atom from substrate, initiating the conversion of substrate to product. In order to accomplish this radical chemistry, AdoMet radical enzymes typically house the [4Fe-4S] cluster and bound AdoMet in a full or partial triose phosphate isomerase (TIM) barrel fold (Figure 2).^4^ The cluster is bound by a CX_3_CX_2_C motif, although exceptions to the spacing of the cysteines are not unusual. This AdoMet radical domain can be combined with domains known for binding other cofactors and specific substrate functional groups such as a cobalamin (Cbl) binding domain, domains for additional Fe–S clusters, or domains for recognizing peptide substrates in what has been labeled a “plug and play” strategy to account for the diversity in chemical reactions performed by this superfamily.^2^

A large subgroup within the superfamily is made up of enzymes that have a Cbl-binding domain, and enzymes within this subgroup are the focus of this Perspective. An estimate of the size of this subgroup from the Structure–Function Linkage Database in 2017 included about 7000 members.^12^ More recently, a new collection of sequences from RadicalSAM.org places the subgroup size at closer to 10 000 members.^13^ What constitutes a Cbl-binding domain was determined when Cbl was first visualized bound to a protein through an X-ray crystal structure of a fragment of methionine synthase (MetH).^14^ Cbl is held within a Rossmann fold domain (Figure 2). The dimethylbenzimidazole (DMB) moiety that acts as a lower ligand to the cobalt in free Cbl is displaced by a histidine (His) residue in the case of MetH − so-called base-off, His-on coordination. The first two structures of Cbl-dependent AdoMet radical enzymes both have different features in the lower axial ligand site.^15,16^

Cbl-dependent enzymes are chiefly associated with radical chemistry and methylation reactions.^17^ The Cbl cofactor can fluctuate between the cob(I)alamin, cob(II)alamin, and cob(III)alamin states depending on whether an upper axial ligand is bound to the cobalt. AdoMet is also a known methyl group donor and initiator of radical chemistry in conjunction with the [4Fe-4S] cluster, so Cbl-dependent AdoMet radical enzymes house two seemingly redundant cofactors.^18^ The standard mechanism for methylation of a substrate in these enzymes involves two AdoMet molecules and Cbl as a methyl transfer station (Figure 1).^19^ First, one molecule of AdoMet is attacked by the cob(I)alamin nucleophile to transfer the methyl group from the sulfur atom to the cobalt to make methylcob(III)alamin and *S*-adenosyl-l-homocysteine (AdoHcy). The second molecule of AdoMet is used in classic AdoMet radical chemistry as described earlier to produce a substrate radical. This substrate radical then abstracts the methyl group from methylcob(III)alamin resulting in homolytic cleavage of the cobalt–carbon bond and methylation of the substrate at a non-nucleophilic atom.

Studying Cbl-dependent AdoMet radical enzymes requires overcoming several major challenges. For one, many of these enzymes are involved in natural product pathways with complex substrates or substrates that have yet to be structurally elucidated. These natural product Cbl-dependent AdoMet radical enzyme reactions are summarized in a recent review by Wang.^20^ Insolubility or purification with low or no activity is another prevalent technical challenge for this enzyme subgroup. One recent method of boosting solubility through improving Cbl uptake in *Escherichia coli* has led to a number of enzyme characterization studies.^21^ Lanz et al. were able to improve the yield of pure TsrM, an enzyme involved in antibiotic biosynthesis, from 0.5 mg/L to 3.7 mg/L through coexpression with genes important for Cbl uptake across the outer and inner *E. coli* membranes.^21^ These genes were packaged on a plasmid as part of a new overexpression method for Cbl-dependent AdoMet radical enzymes. These results have been significant for further characterizing TsrM and other previously known enzymes and will be important for taking on other challenging members of the Cbl-dependent AdoMet radical subgroup. Additional breakthroughs in methodology for working with this subgroup are summarized in a recent review by Sinner et al.^22^

Pioneering biochemical and structural studies are shaping our view of the subgroup in exciting and unexpected ways. In this Perspective, we chronicle the decades-long journey to piece together the activity and mechanism of the unusual Cbl-dependent AdoMet nonradical methylase TsrM. This work has most recently culminated in a crystal structure of the enzyme that provides further evidence for its atypical use of AdoMet and a [4Fe-4S] cluster. We also highlight how recent mechanistic studies of radical methylases utilize similar methods as those employed over time on TsrM and how the discovery of new enzymes with the help of bioinformatic analyses is expanding our concept of the subgroup.

## Establishment of TsrM Methylation Activity

**Figure 3:**
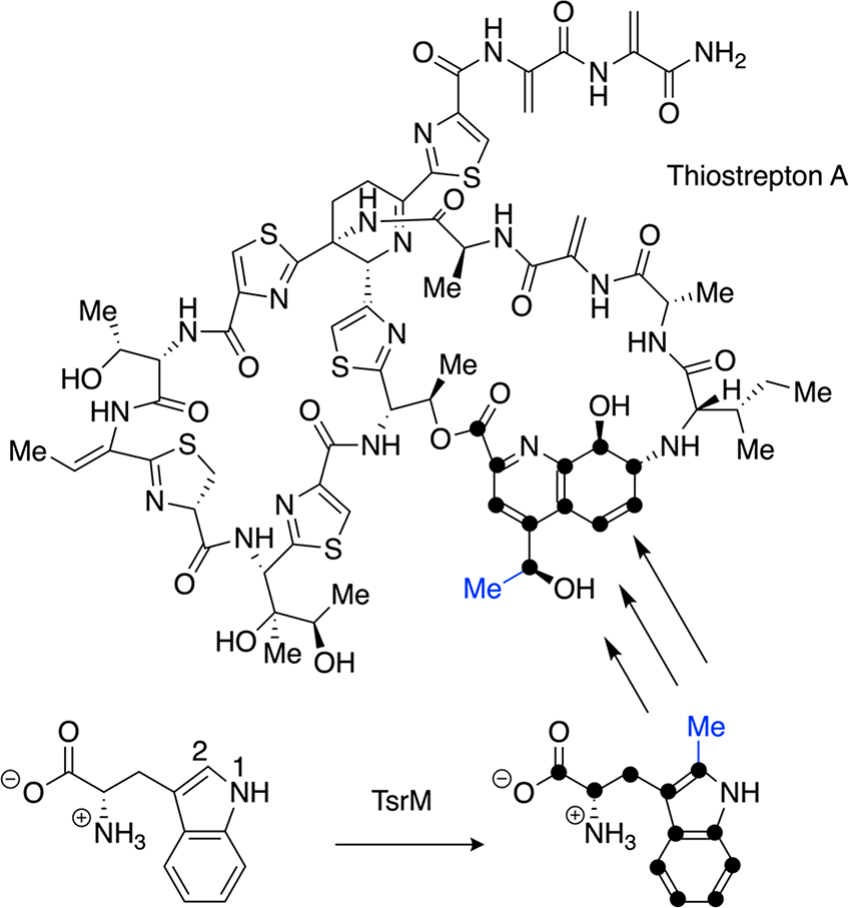
Structure of thiostrepton A and the reaction TsrM performs in its biosynthesis. The methylated Trp forms the quinaldic acid moiety of the final natural product.

The timeline to establish TsrM enzyme activity exemplifies the challenges of working with Cbl-dependent AdoMet radical enzymes. Initial studies around 1990 laid the groundwork for the type of chemistry performed, but a biochemical analysis with purified enzyme took another 20 years to accomplish. TsrM is involved in the biosynthesis of thiostrepton A, a ribosomally synthesized and posttranslationally modified peptide with antibacterial activity.^23,24^ Thiostrepton A is produced by numerous bacterial species and contains a quinaldic acid moiety with carbon atoms derived from tryptophan (Trp) and Met (Figure 3).^24,25^ A retro-biosynthetic analysis suggested that the carbon from Met likely originated as a methylation at C2 of Trp.^25^ Zhou et al. made a number of conclusions through multiple labeling studies.^25^ First, they determined that Trp methylated at the C2 position is an on-pathway intermediate to thiostrepton A by feeding d,l-2-methyl-[3′-^13^C] Trp to *Streptomyces laurentii* and recovering the ^13^C label in the isolated thiostrepton A product. Next, they established that Trp and AdoMet are the substrates for the methylation reaction since cell-free extracts of an *S. laurentii* culture were capable of catalyzing the methylation of Trp from [methyl-^3^H]-AdoMet. Finally, Met feeding studies with *S. laurentii* were performed to determine that the reaction occurs with net retention of configuration with respect to the methyl group from Met to the product. The authors speculated that a cofactor such as Cbl could be involved given that net retention of configuration could occur through two inversions of configuration, but isolation of the enzyme could not be achieved to investigate this proposal further.^25^ Additional experiments were performed with cell-free extracts from *S. laurentii* in lieu of obtaining purified enzyme.^26^ These experiments provided more details of the transformation such as the observation that methylation activity also occurred for indolepyruvic acid as a substrate but not indole, as measured by a combination of high performance liquid chromatography (HPLC) analysis and scintillation counting to track the radiolabeled methyl group from AdoMet. The addition of 1-methyl Trp was shown to act as an inhibitor to the formation of 2-methyl Trp, thus providing the first evidence that components in connection to the indole nitrogen are important for the reaction.^26^ Benjdia et al. also performed substrate scope experiments via HPLC analysis with purified TsrM decades later and determined that TsrM is tolerant of substitutions to the indole ring and will accept serotonin as a substrate as well.^27^

Studies with soluble and active TsrM were first published in 2012, demonstrating the necessity of a Cbl cofactor for the reaction.^28^ Liquid chromatography mass spectrometry (LC-MS) analysis revealed that AdoHcy is produced in the TsrM reaction with Trp and AdoMet, which would be expected based on the standard methylation mechanism outlined in Figure 1. In addition, isotopic labeling studies with *S*-adenosyl-[*methyl*-^2^H_3_]methionine (AdoMet-*d*_3_) established that the methyl group is transferred from AdoMet to form methylcob(III)alamin with the three deuterium atoms all migrating to the 2-methyl Trp product.^28^ However, HPLC analysis of the reaction indicated that the standard AdoMet radical enzyme product 5′-dAdo was not produced.^28^ Therefore, the methyl group transfer from AdoMet to the Cbl cofactor appears to be equivalent to other members of the Cbl-dependent AdoMet radical subgroup, but the method in which the methyl group transfers from Cbl to the substrate could not be readily determined.

## Mechanistic Studies Indicate TsrM Is a Nonradical Methylase

Spectroscopic analyses were performed by Blaszczyk et al. to interrogate the AdoMet radical machinery of TsrM.^29^ TsrM has a canonical CX_3_CX_2_C sequence motif for coordinating a [4Fe-4S] cluster. Mössbauer spectroscopy indicated that the [4Fe-4S] cluster has a unique iron that may bind an oxygen or nitrogen ligand. However, hyperfine sublevel correlation (HYSCORE) spectroscopy was used to determine that AdoMet does not associate with the [4Fe-4S] cluster with its carboxylate and amino moieties as would be expected based on other AdoMet radical enzymes.^4,29^ Addition of the Trp substrate did not perturb the Mössbauer spectrum and was ruled out as directly coordinating to the cluster.^29^ Separately, the authors performed experiments to examine further the observation that 5′-dAdo is not produced.^29^ A subset of AdoMet radical enzymes recycle AdoMet in the reaction by regenerating it from 5′-dAdo and Met after hydrogen atom abstraction from the substrate.^1^ If this regeneration reaction was occurring, then performing the enzyme assay with Trp-*d*_8_ would result in deuterium incorporation into AdoMet. LC-MS analysis confirmed that no deuterium enrichment took place for either AdoMet or AdoHcy, thus providing additional evidence that TsrM does not perform any previously known AdoMet radical chemistry. Therefore, the identity of the species coordinating the unique iron and the overall function of the AdoMet radical machinery in TsrM were left unresolved.

A radical methylation mechanism distinct from that shown in Figure 1 was initially proposed by Benjdia et al. based on UV–visible spectroscopic evidence for the presence of a cob(II)alamin species and to account for the lack of formation of 5′-dAdo.^27^ If cob(II)alamin is generated in the reaction, it would indicate homolytic bond cleavage of the cobalt–carbon bond. Electron paramagnetic resonance (EPR) spectroscopy performed by Blaszczyk et al. also suggested a five-coordinate cob(II)alamin is associated with TsrM, and this experiment went further to ascertain that the cobalt is not coordinated by a nitrogen atom.^29^ Therefore, TsrM appears to bind Cbl in a base-off, His-off orientation distinct from MetH. However, additional UV–visible and EPR spectroscopic analyses by Blaszczyk et al. failed to support the accumulation of cob(II)alamin during TsrM turnover.^30^

**Figure 4:**
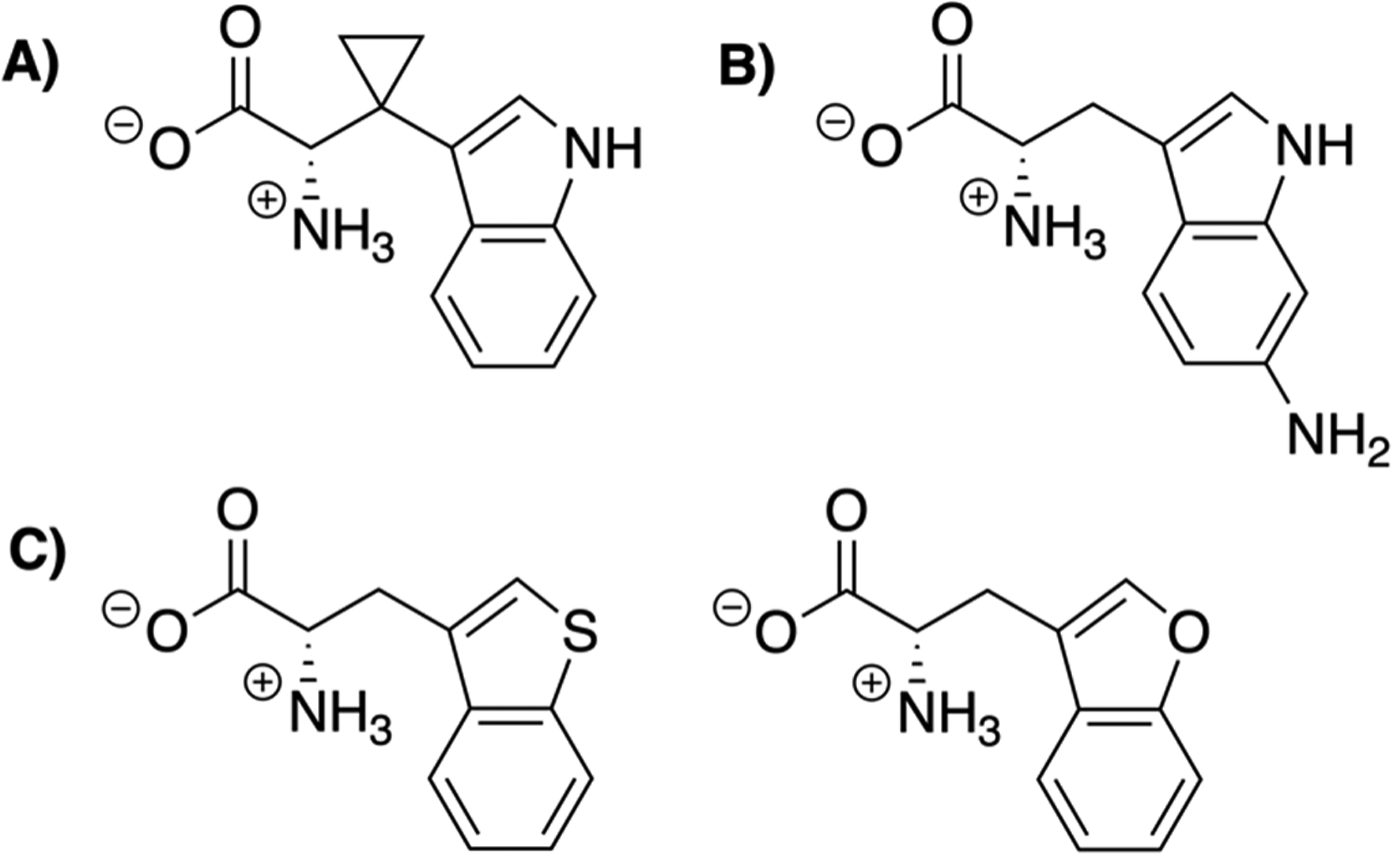
Substrate analogues used to study the TsrM mechanism. (A) A β-cyclopropyl-Trp analogue was used as a radical trap probe. (B) 6-Amino-Trp was employed as a radical-stabilizing analogue. (C) 1-Thia-Trp and 1-oxa-Trp were used to investigate the importance of the N1 amine.

A radical trap probe (β-cyclopropyl-Trp, Figure 4) was also employed by Blaszczyk et al. to examine the possibility of a radical mechanism.^30^ LC-MS analysis indicated that TsrM does accept this substrate analogue, and nuclear magnetic resonance (NMR) spectroscopy was used to clearly define the product as methylated β-cyclopropyl-Trp with no detected formation of the ring-opened product. The radical could still be too short-lived to be trapped, but the authors also did not observe substrate radical formation by EPR spectroscopy on a substrate analogue (6-amino-Trp, Figure 4) that would provide a radical-stabilizing effect.^30^ Additionally, Blaszczyk et al. established that TsrM can turn over substrate without a reductant that would traditionally be added to reduce the [4Fe-4S] cluster in AdoMet radical chemistry.^31^ TsrM did perform better with a flavodoxin/flavodoxin reductase/NADPH reducing system, but the authors determined that NADPH is not consumed during each turnover. To investigate the use of NADPH further, TsrM was preloaded with methylcob(III)alamin (since the enzyme was not isolated with this form of Cbl), and this form of TsrM achieves the same initial turnover numbers with or without the reducing system. These results indicate that the reducing system may play a role in converting oxidized Cbl states to cob(I)alamin so that AdoMet can form methyl(III)cobalamin and move forward with the reaction.^31^ Additional work will be required to determine if the [4Fe-4S] cluster shuttles the reducing equivalents to Cbl. Otherwise, the role of the [4Fe-4S] cluster in TsrM is still unknown.

**Figure 5:**
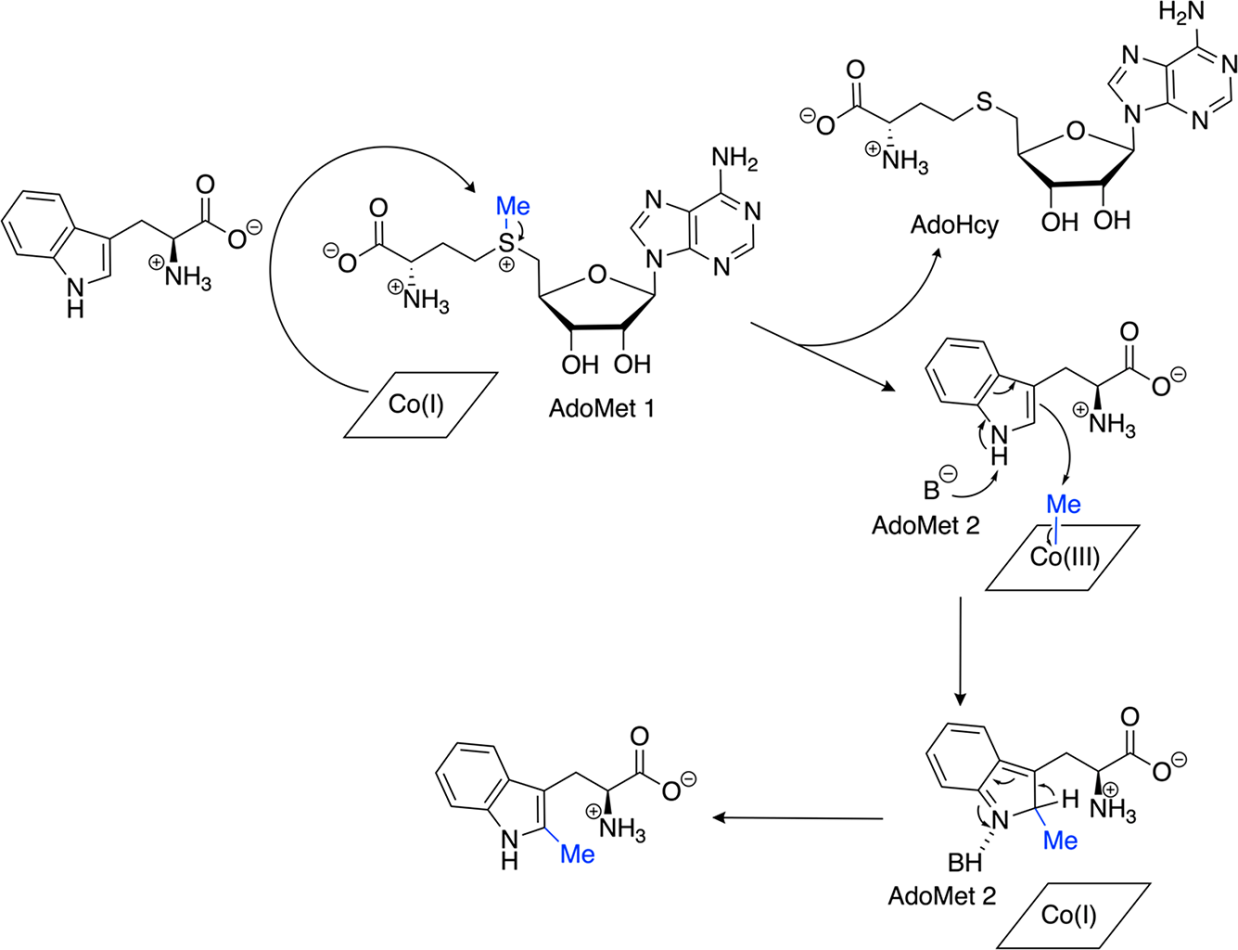
Proposed nonradical mechanism for TsrM methylation of Trp. This mechanism is similar to the radical mechanism in Figure 1 in that one molecule of AdoMet is used to methylate cob(I)alamin and a second molecule of AdoMet is proposed to be involved. The second AdoMet acts as a base to activate the C2 nucleophile for attack on methylcob(III)alamin.

With minimal evidence for a radical mechanism, Blaszczyk et al. began investigating the possibility of a polar mechanism involving deprotonation of the N1 amine of Trp to kick off a nucleophilic attack on methylcob(III)alamin to methylate Trp at the C2 position (Figure 5).^30^ TsrM does not tolerate substrate analogues that alter the N1 amine either through methylation^26,27^ or conversion to 1-thia-Trp or 1-oxa-Trp (Figure 4).^30^ The last two substrate analogues reportedly still produced methylated product, but only at the limit of detection for the LC-MS/MS experiments.^30^ Therefore, the most recent proposal to account for the biochemical data consists of nonradical methylation at C2 with the N1 position playing a key role in setting up a nucleophilic attack on methylcob(III)alamin.^30^ TsrM is proposed to perform the first part of the standard methylation mechanism from Figure 1 that includes cob(I)alamin attack of AdoMet to generate methylcob(III)alamin and AdoHcy. The methyl group is transferred to substrate through heterolytic cleavage of the cobalt–carbon bond via the action of a catalytic base that deprotonates the N1 of the indole ring and the attack of the nucleophilic C2 on the methyl group (Figure 5). This proposal generates questions including: how would the enzyme facilitate the nucleophilic attack on methylcob(III)alamin, and what is the identity of the catalytic base needed to deprotonate Trp?

## Crystal Structure of TsrM Provides Context for Atypical AdoMet Radical Behavior

**Figure 6:**
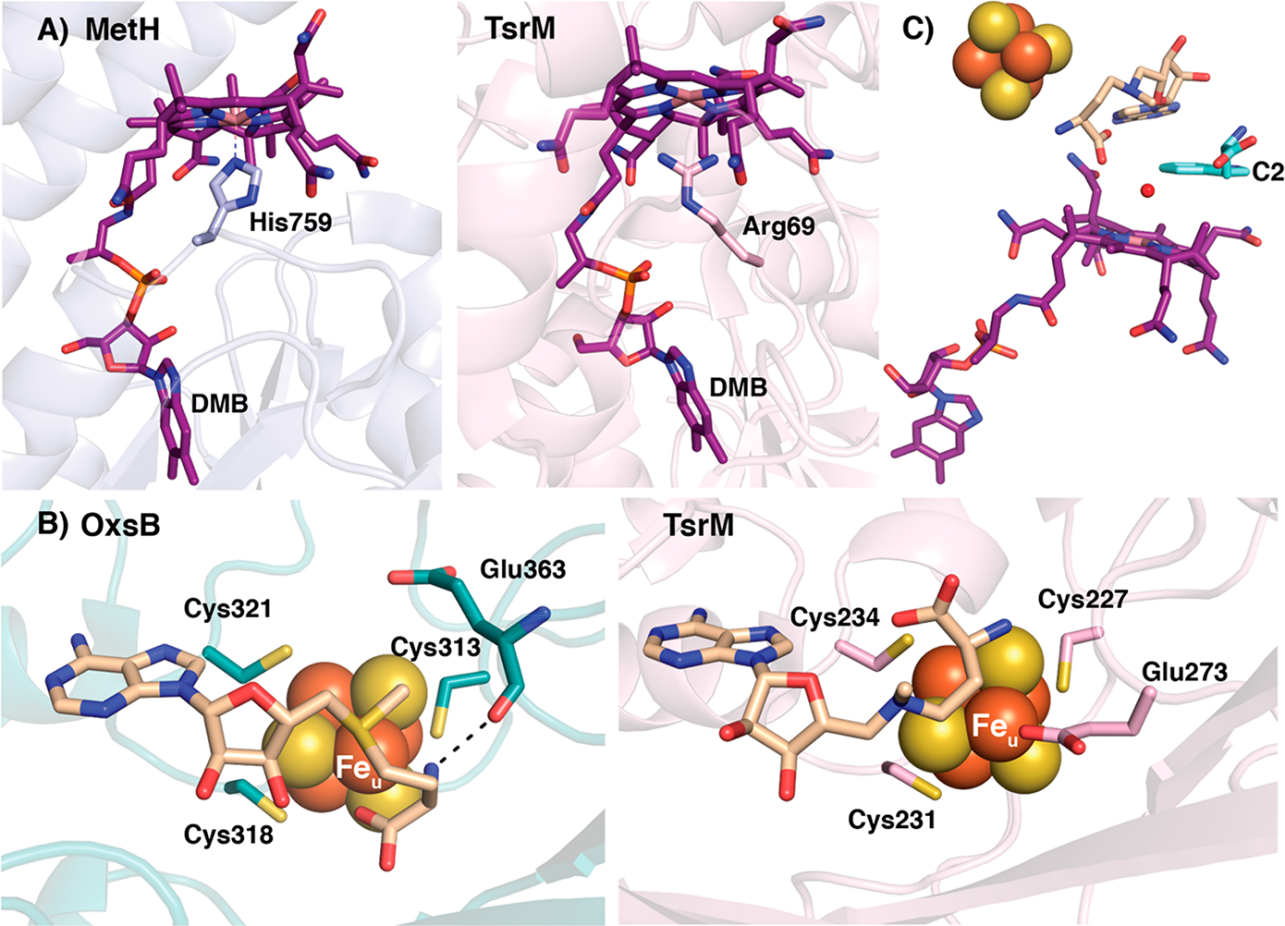
Views of the TsrM active site cofactors and substrate positioning in comparison to MetH and OxsB. (A) The TsrM Cbl is bound in a base-off/His-off mode without anything coordinating the cobalt in the lower axial ligand position. The closest residue is Arg69. A comparison to MetH Cbl binding with the base-off/His-on mode is shown on the left. DMB = dimethylbenzimidazole. (B) The TsrM [4Fe-4S] cluster is bound by a CX_3_CX_2_C motif with the unique iron (labeled Fe_u_) coordinated by Glu273. The derivative aza-AdoMet is bound near the cluster but not in a coordinating position. AdoMet coordination to the Fe_u_ of the OxsB [4Fe-4S] cluster is shown on the left. Glu363 of OxsB is in a similar position as Glu273 of TsrM. Glu363 does not ligate the cluster, but its backbone carbonyl is in hydrogen bonding distance (2.9 Å) to the amino group of AdoMet. (C) The overall placement of the two cofactor machineries of TsrM with the Trp substrate in the middle. The C2 position that is methylated in the product is labeled. The [4Fe-4S] cluster is in orange and yellow, Cbl is in deep purple, aza-AdoMet and AdoMet are in wheat, water (2.8 Å from Co) is in red, and Trp is in aquamarine. MetH PDB ID: 1BMT; TsrM PDB ID: 6WTF; OxsB PDB ID: 5UL4.

The structure of TsrM and additional experiments provide evidence for how this nonradical mechanism could be accomplished.^16^ TsrM has a Rossmann fold for binding Cbl, a partial TIM barrel for binding the [4Fe-4S] cluster and AdoMet, and a C-terminal domain (Figure 2). Knox et al. also determined a structure of TsrM containing Cbl, the [4Fe-4S] cluster, Trp, and aza-AdoMet that highlights the active site architecture with the substrate positioned in between the cofactor machineries (Figure 6).^16^ However, Knox et al. noted that Trp is likely not in the conformation needed for catalysis as the C2 position is pointing away from the Cbl and is too far (7.0 Å) from the cobalt for methylation. The derivative aza-AdoMet with a nitrogen instead of a sulfur atom was used in the crystallization to prevent turnover. As predicted by spectroscopic methods, TsrM deviates from MetH in binding Cbl both base-off and His-off (Figure 6A). An arginine (Arg) residue sits in the lower axial position without coordinating the cobalt but close enough to the cobalt (3.4 Å) to prevent water ligation (Figure 6A, right panel). The lack of a lower ligand weakens the C–Co bond of methylcob(III)alamin, which is in a destabilized penta-coordinate state, making the methyl moiety a better target for the weaker C2 nucleophile of Trp.^16^ This Arg is conserved for all TsrM orthologs and substitution to lysine destroys Trp methylation activity, although methylation of cob(I)alamin still occurs.^16^ The fact that the first methyl transfer from AdoMet to cob(I)alamin is preserved further supports the use of Arg in facilitating the specific event of Trp methylation.

Elements of [4Fe-4S] cluster binding are also different for TsrM compared with other AdoMet radical enzymes and provide evidence for AdoMet acting as a catalytic base to deprotonate N1 of Trp as opposed to acting as a radical cofactor.^16^ Although TsrM does contain the canonical CX_3_CX_2_C motif for coordinating the cluster, the unique iron is coordinated by a glutamate (Glu273) instead of AdoMet (Figure 6B, right panel). Notably, the presence of the Glu in this location is common in AdoMet radical enzymes.^4^ OxsB, another structurally characterized Cbl-dependent AdoMet radical enzyme, has a similarly positioned Glu residue (Glu363)^15^ that helps secure AdoMet as a ligand to the cluster through backbone hydrogen bonding as is often observed (Figure 6B).^4^ Thus, TsrM appears to use a residue that is commonly found near the unique iron in a new way. With Glu preventing AdoMet from binding the cluster, AdoMet is not used for radical generation. Instead, in its new location, the carboxylate moiety of AdoMet (aza-AdoMet in the structure) is closer to Trp and Cbl, where it can play a role in acid–base catalysis. Enzyme activity assays with premethylated TsrM containing methylcob(III)alamin confirmed that AdoMet is required for the formation of MeTrp and that using a decarboxylated derivative of AdoMet does not restore activity to wild-type levels.^16^ These structural and biochemical data imply that AdoMet still functions in two roles in the TsrM methylation mechanism even though one role is not the same as in the standard methylation mechanism of Figure 1; instead of being a radical cofactor, AdoMet appears to act as the catalytic base. This example is not the first of a substrate serving as a catalytic acid or base,^32^ but the TsrM case is, as far as we know, the first example of AdoMet playing the role of catalytic base in an AdoMet radical enzyme.

The TsrM structural data^16^ thus served to explain many of the more surprising biochemical findings that were described above. For example, from the structure, we can now understand why TsrM does not form the 5′-dAdo• species upon AdoMet binding; Glu273 blocks AdoMet coordination of the unique Fe. We can also rationalize how TsrM weakens the methyl-Co bond for Trp methylation; Arg69 blocks ligation of the Co at its lower axial position, preventing formation of the more stable six coordinate methylcob(III)alamin species that is found in other cobalamin-dependent methylases like MetH. Finally, as described above, the structural data suggest the identity of the catalytic base: AdoMet.

This being said, there are important structural snapshots that are missing. We already noted that Trp is not positioned with its C2 carbon pointing toward the cobalamin. Additionally, aza-AdoMet appears positioned for a role as a catalytic base but not for its role in methylation of cob(I)alamin. With AdoMet bound as shown in Figure 6, the distance between the methyl moiety of AdoMet and the Co would be over 7 Å, too far for an S_N_2 methyl transfer. Interestingly, a crystal structure depicting the activation complex of MetH with AdoHcy bound, also shows a long distance (∼7 Å) between the sulfur of AdoHcy and the Cbl Co.^33^ Thus, all TsrM and MetH structures that are currently available fail to reveal the positioning of AdoMet that is required for cob(I)alamin methylation. In the case of TsrM, the Trp substrate would appear to block closer positioning of AdoMet and Cbl, and in MetH, a Tyr side chain appears to be in the way. Therefore, more data are needed for us to fully understand cobalamin methylation by AdoMet, a reaction that is likely to be step 1 for all members of the Cbl-dependent AdoMet radical methylase superfamily.

## Recent Mechanistic Work on Cbl-Dependent AdoMet Radical Methylases

**Figure 7:**
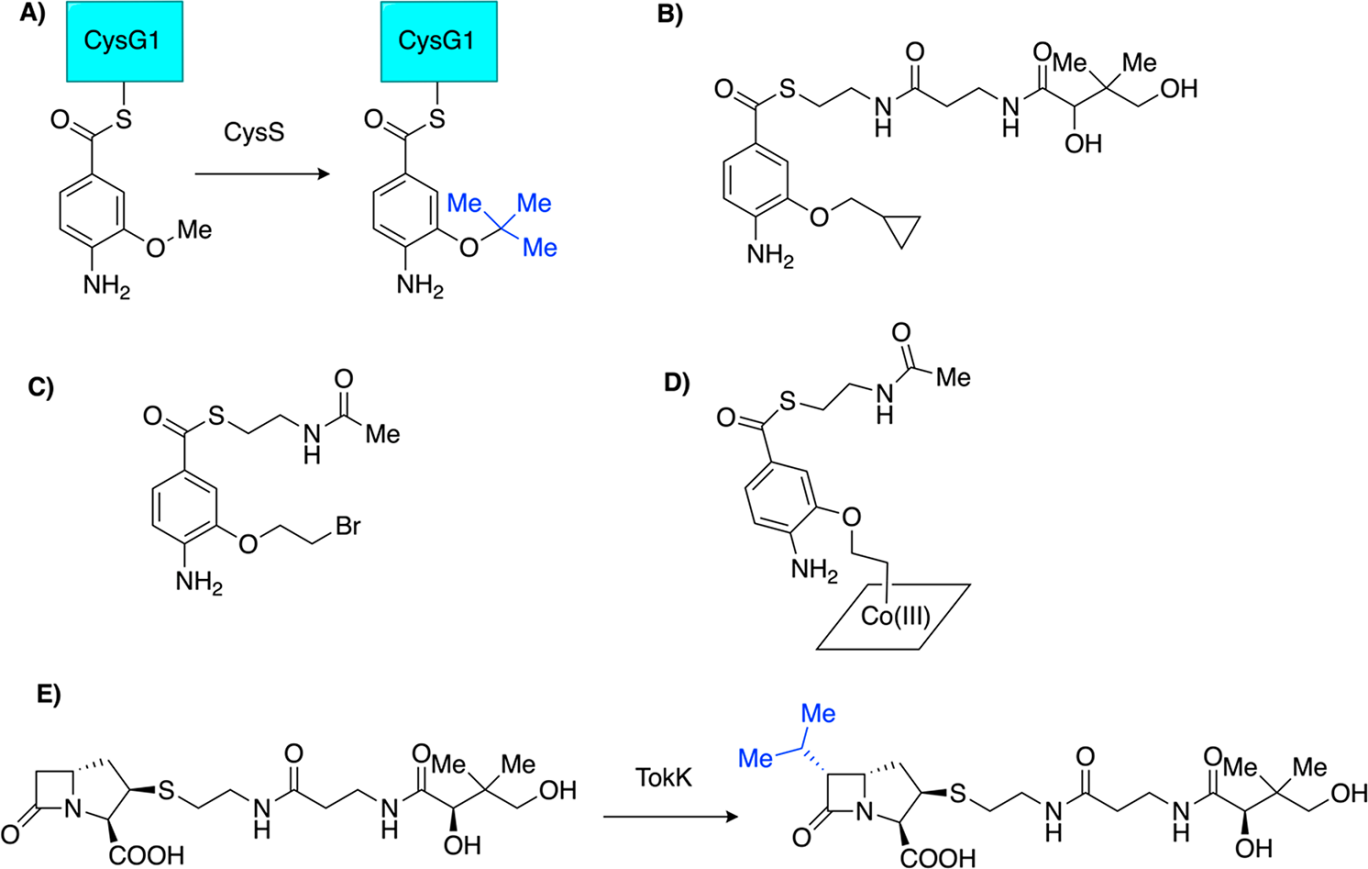
Reactions of CysS and TokK that perform multiple methylations on the same substrate and substrate analogues used to study the mechanism of CysS. (A) The CysS reaction produces a *tert*-butyl group. (B) A cyclopropyl radical trapping analogue of the CysS substrate also eliminates the need to use the CysG1 peptidyl carrier protein for enzyme assays. (C) A bromoethoxy-substituted analogue was also employed to investigate the radical mechanism of CysS. (D) The chemical cross-link that forms between the bromoethoxy-substituted analogue and Cbl. (E) The reaction of TokK consists of multiple methylations to form an isopropyl group.

Techniques used through the decades to study TsrM are still prevalent in more recent mechanistic studies of other Cbl-dependent AdoMet radical methylases. We point out recent studies on CysS, TokK, and Fom3 that take advantage of the classic mechanistic enzymology tools of radical trapping probes and isotope labeling, as well as advances in ultraperformance liquid chromatography-high resolution mass spectrometry (UPLC-HRMS) and reaction rate modeling. CysS acts within a nonribosomal peptide synthetase biosynthetic pathway to form the antibacterial compound cystobactamid.^34^ CysS performs multiple methylations on its 3-methoxy-4-aminobenzoic acid substrate scaffolded on a peptidyl carrier protein (Figure 7A).^35^ Wang et al. demonstrated that a synthetic mimic of the substrate was suitable for biochemical studies and would circumvent difficulties working with the peptidyl carrier protein (Figure 7).^35^ Wang and Begley were able to proceed with radical trapping experiments that allowed for estimation of the rate of methyl transfer and characterization of an unexpected intermediate.^36^ These results led to a revised proposal for the active site architecture.^36^ CysS is proposed to proceed through the standard methylation mechanism outlined in Figure 1. Substrate analogues containing a cyclopropyl ring and a bromoethoxy moiety (Figure 7B, C) were designed to trap the substrate radical formed by 5′-dAdo• hydrogen atom abstraction at the methoxy substituent and thus estimate the rate of methyl transfer from methylcob(III)alamin to the substrate. Using the cyclopropyl analogue, a comparison was made between the amount of methylated product (the major product) and the amount of products resulting from ring opening.^36^ To solve for the rate of methyl transfer, the authors used the known rate for nonenzymatic ring opening of a (methoxymethyl)-cyclopropyl radical, leading to a methyl transfer rate of 2.4 × 10^8^ s^–1^.^36,37^ This value is significantly higher than the methyl transfer rate for MetH (140 s^–1^) when transferring a methyl group from methylcob(III)alamin to its homocysteine substrate.^38^ Wang and Begley cautioned that their value should be considered an upper limit for the CysS reaction given their estimation of the rate of ring opening for their enzyme-bound substrate with extra radical stabilization provided by an aryl group to be the same as that of the smaller (methoxymethyl)-cyclopropyl radical. In any case, the authors have taken a necessary step in performing radical trapping experiments on a Cbl-dependent AdoMet radical enzyme to gain insight into methyl transfer rates.

A bromoethoxy substrate analogue was also prepared to provide additional evidence for the CysS rate of methyl transfer, but an unexpected product materialized as the substrate analogue cross-linked with the cob(I)alamin cofactor (Figure 7D). This product was extensively characterized by LC-MS/MS, MS and UV analysis in comparison to a synthetic standard, and by derivatization experiments based on expected reactivity of the cross-linked compound.^36^ Cross-linked product formation also was not strongly inhibited by AdoMet, indicating that cross-linking is faster than methylation by AdoMet. The authors therefore proposed two conformations for AdoMet − one places the methyl group in a position for transfer to cob(I)alamin and the other in a position to avoid clashes with methylcob(III)alamin.^36^ AdoMet sampling of this second binding mode before methylation of cob(I)alamin would account for observation of the cross-linked product with the bromoethoxy substrate analogue. This proposal of two binding conformations for AdoMet aligns with structural data from the ring-contracting Cbl-dependent AdoMet radical enzyme OxsB where two conformations of AdoMet were visualized,^15^ and with the TsrM structure that shows aza-AdoMet positioned for its role as a catalytic base, but not for its role of a methylating agent of cobalamin (see above).^16^ The occurrence of the cross-linked product also suggests that the CysS substrate sits in between the Cbl and AdoMet radical machinery poised for action at the cobalt center. This mechanistic conclusion is consistent with Trp positioning observed in the TsrM crystal structure and predicted substrate positioning based on the OxsB crystal structure.^15,16^

TokK catalyzes three sequential methylations (like CysS) along the biosynthetic pathway to the carbapenem compound asparenomycin (Figure 7E).^39^ Sinner et al. relied on the Cbl uptake system described earlier for TsrM^21^ to produce TokK and confirmed its role in all three methylations by comparing the enzymatic product to a synthetic standard.^39^ Interestingly, two other genes in the asparenomycin gene cluster are annotated as Cbl-dependent AdoMet radical enzymes, but the function of these two putative subgroup members is unknown.^39^ To further characterize the multiple methylations of TokK, time course experiments were performed to compare the relative rates of methylation between TokK and another carbapenem Cbl-dependent AdoMet radical methylase (ThnK) that accepts the same substrate but only performs two methylations to form an ethyl substituent en route to thienamycin.^40^ The individually methylated intermediates from both TokK and ThnK were detected by UPLC-HRMS and a kinetic model and simulation curves were created with the tools COPASI and VCell.^39,41,42^ The authors concluded that TokK performs its first and second methylation events 2-fold and 10-fold faster than ThnK based on modeling of the experimental data to obtain relative rate constants.^39^ The second TokK methylation rate constant is estimated to be a little over 2-fold faster than the first TokK methylation, although the rate of the third methylation drops off significantly. Finally, the fact that individually methylated intermediates could be detected provides evidence for a sequential enzyme mechanism.^39^

**Figure 8:**
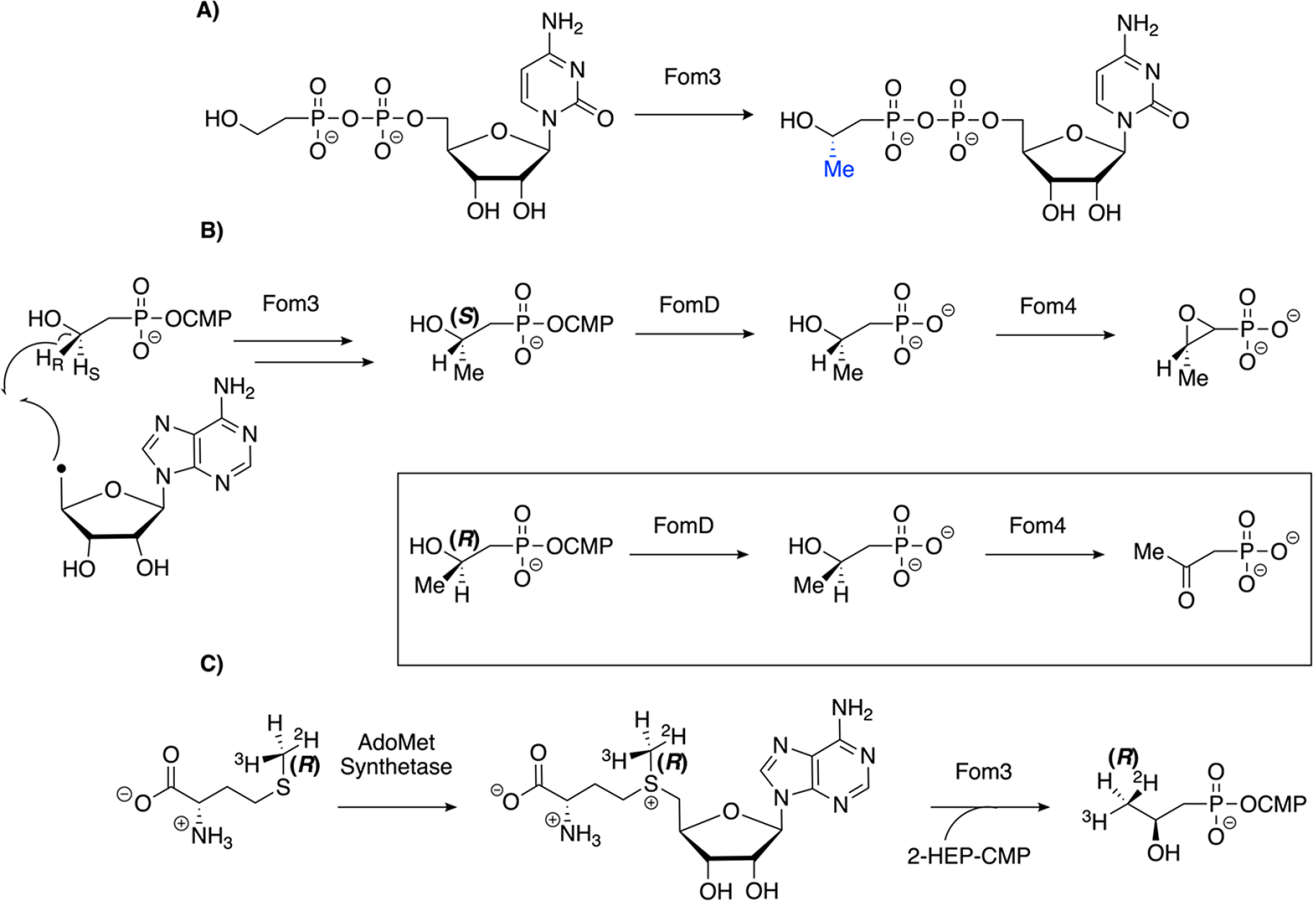
Reactions important to the study of Fom3. (A) Fom3 converts 2-HEP-CMP into (*S*)-2-HPP-CMP. (B) The experimental setup to determine that inversion of configuration occurs in relation to the methylated carbon during the Fom3 mechanism when the 5′-dAdo• abstracts the pro-*R* hydrogen atom to form the (*S*)-2-HPP-CMP product. The stereochemistry of the product was confirmed using the activities of FomD and Fom4 to distinguish between (*R*)-2-HPP and (*S*)-2-HPP. (C) Feeding studies and enzymatic studies with isotopically labeled Met established that the reaction occurs with overall retention of configuration in relation to the methyl group.

Mechanistic work on Fom3 has exploded in the past few years after addressing both challenges of determining the substrate and obtaining decent quantities of soluble enzyme.^21,43^ Researchers have now been able to investigate details of the stereochemistry of the reaction and implications for the active site architecture. Fom3 is involved in the biosynthesis of fosfomycin, a clinically used antibiotic, from *Streptomyces* species.^44−46^ The substrate (5′-cytidylyl)-2-hydroxyethylphosphonate (2-HEP-CMP) is converted to (5′-cytidylyl)-2-hydroxypropylphosphonate (2-HPP-CMP, Figure 8A).^43^ Soluble enzyme with improved Cbl incorporation and higher activity was obtained by expressing the Cbl uptake genes during production of the enzyme.^21^ Three research groups addressed the issue of reaction stereochemistry at the methylated carbon for Fom3 from two different *Streptomyces* species by preparing isotopically labeled substrate analogues deuterated at the pro-*R* and pro-*S* position on C2.^47−49^ Analysis of enzyme assay products by LC-MS techniques showed that deuterium is incorporated into 5′-dAdo when the substrate is labeled at the pro-*R* position on C2 (Figure 8B). To examine the stereochemistry of the product, Sato et al. used chiral ligand exchange chromatography to separate (*S*)- and (*R*)-2-HPP-CMP, but only (*S*)-2-HPP-CMP was observed.^47^ Wang et al. and McLaughlin and van der Donk instead took advantage of the ability of the downstream biosynthetic enzyme Fom4, which can distinguish between (*R*)-2-HPP and (*S*)-2-HPP.^48,49^ Fom4 catalyzes the last step in fosfomycin biosynthesis after FomD removes the CMP group (Figure 8B).^49,50^ Fom4 converts (*S*)-2-HPP to fosfomycin and (*R*)-2-HPP to (2-oxopropyl)-phosphonate.^51^ Wang et al. used acid hydrolysis on the Fom3 product to separate HPP from CMP.^48^ McLaughlin and van der Donk used FomD to accomplish the same goal so that Fom4 could then act on HPP.^49^ Analysis of the Fom4 product by ^31^P NMR spectroscopy revealed fosfomycin as the only product, indicating that only (*S*)-2-HPP-CMP was produced by Fom3. Therefore, all authors came to the same conclusion that the Fom3 reaction occurs with inversion of configuration at the methylated carbon based on removal of the pro-*R* hydrogen atom and generation of the (*S*)-2-HPP-CMP product.

Multiple authors make the statement that inversion of configuration at the substrate non-nucleophilic carbon implies an active site geometry in which the substrate sits with the Cbl and AdoMet radical machinery on opposite sides.^19,47−49^ This arrangement is in agreement with proposals made following evidence from the CysS cross-linking results and the visualization of substrate positioning in TsrM.^16,36^ Inversion of stereochemistry upon methylation is also apparent in subgroup members GenD1 and MoeK5 involved in gentamicin C_1_ and moenomycin biosynthesis, respectively, as determined by the chirality of the carbon centers before and after methylation.^52,53^ Only GenK, which is also involved in gentamicin C_1_ biosynthesis, has been reported to proceed via retention of configuration at the methylated carbon based on experiments similar to those for Fom3.^54^ It should be noted that there are far more members of this enzyme family uncharacterized than characterized. Thus, it is too early to say whether GenK will be an outlier when all is said and done.

Finally, two groups continued further with investigation of Fom3 stereochemistry to establish net retention of configuration relative to the methyl group. McLaughlin et al. investigated the in vitro reaction and Schweifer and Hammerschmidt probed the in vivo reaction.^55,56^ Both groups used Met with defined stereochemistry at the methyl group through isotopic labeling with ^2^H and ^3^H termed (*methyl-R*)-Met or (*methyl-S*)-Met. The stereodefined Met molecules were either used to enzymatically generate the AdoMet added to Fom3 reaction mixtures or used in feeding studies with fosfomycin producer *Streptomyces fradiae*. These feeding studies were in line with initial work on TsrM mentioned previously.^25^ The (*S*)-2-HPP-CMP product (in vitro) or fosfomycin (in vivo) carrying the methyl group with defined stereochemistry was then converted to chiral acetate. The stereochemistry of the chiral acetate was determined using the method of Cornforth and Arigoni through enzymatic production of malate.^57,58^ The starting chiral acetate configuration was deduced from analysis of the ^3^H content of the final malate product. For both the in vitro and in vivo experiments, chiral acetate originating from (*methyl-R*)-Met had the *R* configuration, and the use of (*methyl-S*)-Met resulted in chiral acetate with the *S* configuration.^55,56^ Retention of product methyl group configuration relative to Met methyl group configuration is consistent with the proposed general mechanism in Figure 1 which includes inversion of stereochemistry during nucleophilic attack and transfer of the methyl group from AdoMet to methylcob(III)alamin and a second inversion of stereochemistry upon radical recombination during transfer from methylcob(III)alamin to substrate. This stereochemical outcome has also been confirmed via in vivo feeding studies for the production of thienamycin involving ThnK.^59^

## Characterization of Additional Subgroup Members

**Figure 9:**
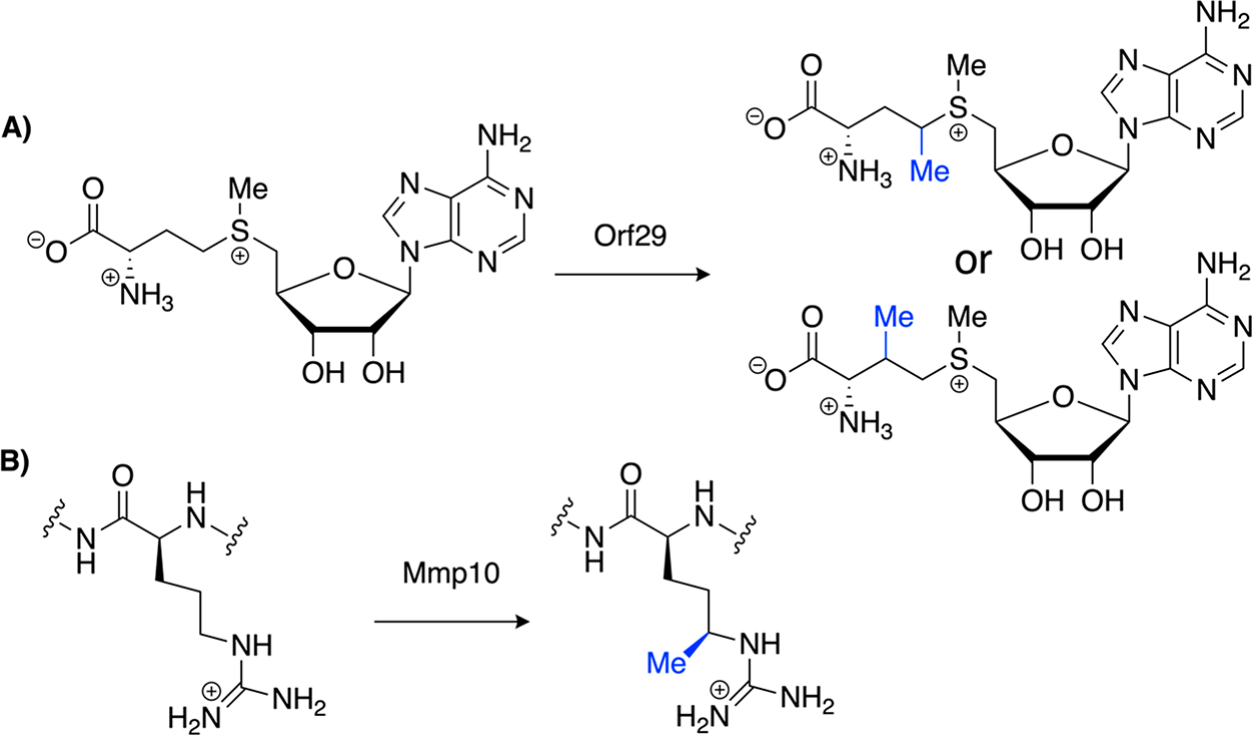
Reactions of recently characterized Cbl-dependent AdoMet radical enzymes. (A) Orf29 methylates AdoMet. (B) Mmp10 methylates an Arg residue in methyl coenzyme M reductase.

Two recent studies exemplify the use of bioinformatics tools to characterize new subgroup members. Maruyama et al. identified a biosynthetic gene cluster suspected of producing a nonribosomal peptide with a 1-amino-2-methylcyclopropanecarboxylic acid (MeACC) moiety as has been characterized in other natural products.^60−62^ One of the genes was predicted, based on sequence similarity, to encode a Cbl-dependent AdoMet radical enzyme, which the authors tentatively assigned as the methylase responsible for MeACC formation. They were able to produce the enzyme (Orf29) using the recently developed Cbl uptake strategy^21^ and determine that Orf29 methylates AdoMet (Figure 9A) before another enzyme (Orf30) forms the cyclopropyl ring to complete MeACC biosynthesis.^60^ Therefore, Orf29 appears to use AdoMet as a substrate and cosubstrate. The authors acknowledge that further characterization is needed to verify the exact position of AdoMet methylation by Orf29.^60^ Future structural characterization would also be valuable for our understanding of how an active site can be designed to accommodate AdoMet as a methyl donor, radical generator, and substrate.

An enzyme involved in posttranslational modification of methyl coenzyme M reductase (MCR) and the production of methane unexpectedly joined the Cbl-dependent AdoMet radical enzyme subgroup after biochemical characterization revealed Cbl as essential for the methylation of an Arg *sp*^3^-hybridized carbon (Figure 9B).^63^ In contrast to the identification of Orf29 with its predicted Cbl-binding domain, methanogenic marker protein 10 (Mmp10) was only identified as an AdoMet radical enzyme superfamily member based on homology of its AdoMet radical domain.^64^ The known Cbl-binding domain was not recognized on Mmp10. Instead, Mmp10 contains a region with homology to a domain found in other proteins and labeled as domain of unknown function 512 (DUF512).^63^ Unfortunately, none of the other proteins in this group have been characterized to see if any connection to Cbl is apparent. Regardless of Mmp10 joining the DUF512 group or not, this enzyme signifies an expansion of the Cbl-dependent AdoMet radical enzyme subgroup. Radle et al. performed a number of biochemical assays to support Cbl usage in the reaction and provide evidence that Mmp10 follows the standard methylation mechanism proposed for other subgroup members (Figure 1).^63^ After observing very little turnover of the Arg-containing peptide surrogate substrate (in place of the full MCR substrate), Mmp10 activity was tested after adding either OHCbl or methylcob(III)alamin to the reaction or the reaction was performed with Mmp10 reconstituted with OHCbl. In both cases, all expected reaction products associated with the mechanism in Figure 1 were generated (MeArg peptide product, AdoHcy, Met, and 5′-dAdo). Radle et al. also performed labeling studies with AdoMet-*d*_3_ as a cosubstrate in the enzyme reaction.^63^ They determined by LC-MS that the methyl group became associated with the MeArg peptide product with retention of all three deuterium atoms, which provided more evidence in support of Mmp10 following the mechanism in Figure 1. This result is distinct from other classes of AdoMet radical methylases that do not involve Cbl but instead have a proposed mechanism that would result in a product with less than three deuterium atoms when using AdoMet-*d*_3_.^63,65^ To provide evidence that Cbl is acting as an intermediate methyl transfer agent, the authors set up two reactions with Mmp10 with reconstituted Cbl that had already been converted to methylcob(III)alamin.^63^ One reaction contained AdoMet and the other reaction contained AdoMet-*d*_3_. Formation of methylcob(III)alamin-*d*_3_ and MeArg-*d*_3_ peptide were both monitored by LC-MS techniques. The concentration of methylcob(III)alamin-*d*_3_ and MeArg-*d*_3_ only grew over time in the reaction with AdoMet-*d*_3_. The concentration of unlabeled methylcob(III)alamin stayed constant in the reaction with unlabeled AdoMet and decreased sharply in the reaction with AdoMet-*d*_3_ once the methylcob(III)alamin from the enzyme was exhausted. These results indicate that the methyl group from AdoMet is transferred to cob(I)alamin and methylcob(III)alamin transfers its methyl group to the peptide substrate as predicted in the mechanism in Figure 1. Future structural analysis of Mmp10 will help to clarify how the unexpected Cbl-binding domain interacts with the Cbl cofactor, how this domain is positioned to interface with the AdoMet radical machinery, and whether there are any differences relative to Cbl-dependent AdoMet radical methylases with recognizable Cbl-binding domains.

## Open Questions and Conclusions

Biochemical and structural characterization of the Cbl-dependent AdoMet radical enzyme subgroup has already revealed fascinating chemical transformations and unexpected results even when only a tiny fraction of the subgroup has been investigated. These enzymes have two cofactor systems that are both capable of radical chemistry and challenging methylations at electrophilic sites. Although the mechanistic diversity of AdoMet radical methylases has already been noted,^65^ housing these two systems within the same active site is bound to turn up even more unexpected chemistry. Finding a nonradical methylase within the Cbl-dependent AdoMet radical enzyme subgroup raises a number of compelling questions. Why TsrM has an AdoMet radical domain to perform its nonradical methylation chemistry remains an open question. Additionally, the current state of the field only allows us to speculate on whether other subgroup members will share a mechanism with TsrM. Another suspected Cbl-dependent AdoMet radical enzyme, CloN6, has been identified through gene deletion studies to methylate an *sp*^2^-hybridized carbon on a pyrrole ring.^66^ However, biochemical studies have not been performed to confirm the necessity of the cofactors or to ascertain a mechanism. In terms of structure, OxsB,^15^ TsrM,^16^ and now TokK^67^ all share the overall scaffold of a Cbl-binding domain and an AdoMet radical domain and yet perform three different mechanisms. Therefore, the details of the active site architecture will be critical for assessing trends in mechanisms. For example, all three of these enzymes have a different residue in the Cbl lower axial ligand space. We have discussed earlier how Arg69 of TsrM appears to aid in the chemistry for nucleophilic attack on methylcob(III)alamin.^16^ Whether the Asn/water ligand for OxsB or the Trp for TokK correlates with the chemistry performed will require more data points from structural information on other subgroup members.^15,67^ A thorough comparison of these three structures is the subject of a recent review by Bridwell-Rabb et al.^68^ Unfortunately, major hurdles exist in protein production and substrate determination for this enzyme subgroup to achieve more biochemical and structural coverage. Efforts to improve on current methodology will have a positive impact on multiple projects, as evidenced by the study to enhance Cbl uptake in protein overexpression.^21^ Our highlighted discussion of TsrM is a clear example of how efforts in method development, mechanistic characterization, and structural determination all came together to elucidate the only known nonradical Cbl-dependent AdoMet radical enzyme and how it has co-opted its cofactors for polar chemistry. Approaching additional subgroup members with a similar variety of experimental techniques will help establish the true range of capabilities of these incredible catalysts.

Note: Since acceptance of this Perspective, a structure of Cbl-dependent AdoMet radical enzyme Mmp10 has been published.^69^
